# 118 Can we reasonably recommend strength training twice weekly for the general adult population aged 18-64 years to secure optimal body functionality?

**DOI:** 10.1093/eurpub/ckae114.153

**Published:** 2024-09-26

**Authors:** Nina Sjørup Simonsen, Stine Bilde Jensen, Sara Tonni Mogensen, Halfdan Thorsø Skjerning, Julie Sandell Jacobsen, Dea Kejlberg Andelius, Nanna Holt Jessen, Lene Gissel Rasmussen, Sebastian Skejø, Solvej Videbæk Bueno, Knud Ryom, Per Kallestrup, Jeanette Reffstrup Christensen, Rasmus Østergaard Nilsen

**Affiliations:** Research Unit For General Practice, Aarhus, Denmark; Department of Public Health, Aarhus University, Aarhus, Denmark; Department of Public Health, Aarhus University, Aarhus, Denmark; Research Unit For General Practice, Aarhus, Denmark; Research Unit For General Practice, Aarhus, Denmark; Research Centre for Health and Welfare Technology, Programme for Rehabilitation, VIA University College, Aarhus, Denmark; Research Unit For General Practice, Aarhus, Denmark; Department of Public Health, Aarhus University, Aarhus, Denmark; Research Unit For General Practice, Aarhus, Denmark; Research Unit For General Practice, Aarhus, Denmark; Department of Public Health, Aarhus University, Aarhus, Denmark; Research Unit For General Practice, Aarhus, Denmark; Department of Public Health, Aarhus University, Aarhus, Denmark; Research Unit For General Practice, Aarhus, Denmark; Department of Public Health, Aarhus University, Aarhus, Denmark; Department of Public Health, Aarhus University, Aarhus, Denmark; Research Unit For General Practice, Aarhus, Denmark; Department of Public Health, Aarhus University, Aarhus, Denmark; Research Unit For General Practice, Aarhus, Denmark; Research Unit of General Practice, Department of Public Health, University of Southern Denmark, Odense, Denmark; DRIVEN, Institute of Sports Science and Clinical Biomechanics, University of Southern Denmark, Odense, Denmark; Research Unit For General Practice, Aarhus, Denmark; Department of Public Health, Aarhus University, Aarhus, Denmark

## Abstract

**Purpose:**

The objective of the study was to conduct a quality assessment of the scientific literature underlying the Danish Health Authorities (DHA) revised recommendations on physical activity (PA), with focus on the new advice on strength training twice a week to secure optimal body functionality in the adult population aged 18-64 years.

**Methods:**

The study was a scoping review. Reviews conducted by the World Health Organization (WHO), the American and Canadian Health Authorities, and evidence referenced in earlier reports from DHA were included for initial review, as DHA refer to these as base for their PA-guideline revision. Studies included in the reviews were title- and abstract screened to assess whether they investigated PA and/or strength training in a population of adults aged 18-64 years. Studies identified through this screening, were furthermore full text screened, and finally included in a quality assessment, if they investigated a strength training intervention in relation to a measure of body functionality. The quality assessment was inspired by STROBE and the Jadad Scale (for observational studies and RCTs respectively) and extended with an assessment of the study’s research goal compliance (causal or predictive) to evaluate the study’s ability to detect eventual effects of the intervention on body functionality.

**Results:**

A total of 25 out of 879 studies identified in the reviews were selected for quality assessment. Great variations in quality were identified, and many studies (13 out of 25) did not achieve 2/3 of potential quality points. The studies generally support strength training as an effective intervention on body functionality. However, 15 of the 16 RCTs investigated a study population with functional limitations, e.g. populations with musculoskeletal disorders, and most studies were based on a study population in the higher end of the age spectrum 18-64 years.

**Conclusions:**

The literature does not clearly support a recommendation on strength training twice weekly for the general adult population to secure optimal body functionality.

The formulation of PA-guidelines should be based on evidence that 1) aims to draw causal conclusions on the effect of the recommended intervention and 2) investigates a population comparable to the target audience.

**Funding:**

None

